# Structural insight into the substrate-binding mode and catalytic mechanism for MlrC enzyme of *Sphingomonas* sp. ACM-3962 in linearized microcystin biodegradation

**DOI:** 10.3389/fmicb.2023.1057264

**Published:** 2023-02-17

**Authors:** Xiaoliang Guo, Zengru Li, Qinqin Jiang, Cai Cheng, Yu Feng, Yanlin He, Lingzi Zuo, Li Rao, Wei Ding, Lingling Feng

**Affiliations:** ^1^Key Laboratory of Pesticide and Chemical Biology (CCNU), Ministry of Education, College of Chemistry, Central China Normal University, Wuhan, China; ^2^The Institute of Physics, Chinese Academy of Sciences, Beijing, China

**Keywords:** MlrC enzyme, microcystins biodegradation, substrate-binding mode, catalytic mechanism, active center, molecular docking

## Abstract

Removing microcystins (MCs) safely and effectively has become an urgent global problem because of their extremely hazardous to the environment and public health. Microcystinases derived from indigenous microorganisms have received widespread attention due to their specific MC biodegradation function. However, linearized MCs are also very toxic and need to be removed from the water environment. How MlrC binds to linearized MCs and how it catalyzes the degradation process based on the actual three-dimensional structure have not been determined. In this study, the binding mode of MlrC with linearized MCs was explored using a combination of molecular docking and site-directed mutagenesis methods. A series of key substrate binding residues, including E70, W59, F67, F96, S392 and so on, were identified. Sodium dodecane sulfate-polyacrylamide gel electrophoresis (SDS-PAGE) was used to analyze samples of these variants. The activity of MlrC variants were measured using high performance liquid chromatography (HPLC). We used fluorescence spectroscopy experiments to research the relationship between MlrC enzyme (E), zinc ion (M), and substrate (S). The results showed that MlrC enzyme, zinc ion and substrate formed E-M-S intermediates during the catalytic process. The substrate-binding cavity was made up of N and C-terminal domains and the substrate-binding site mainly included N41, E70, D341, S392, Q468, S485, R492, W59, F67, and F96. The E70 residue involved in both substrate catalysis and substrate binding. In conclusion, a possible catalytic mechanism of the MlrC enzyme was further proposed based on the experimental results and a literature survey. These findings provided new insights into the molecular mechanisms of the MlrC enzyme to degrade linearized MCs, and laid a theoretical foundation for further biodegradation studies of MCs.

## 1. Introduction

Due to water eutrophication and global warming, the ecological crisis and public health problems caused by harmful cyanobacterial blooms (HCBs) have become a global environmental issue (Paerl et al., 2011; Paerl and Otten, 2013; Xu et al., 2015; Bullerjahn et al., 2016; Visser et al., 2016; Huisman et al., 2018; Sadhasivam et al., 2019; Sehnal et al., 2019; Ji et al., 2020; Li et al., 2020; Du et al., 2021). During the overgrowth of HCBs, toxin-producing cyanobacteria release various microcystins (MCs) into the water body, with MC-LR, MC-RR, and MC-YR being the most toxic and widely distributed MC variants (Hoeger et al., 2007; Atencio et al., 2008; Zeller et al., 2012; Okogwu et al., 2014; He et al., 2018; Tao et al., 2018; Zhang et al., 2021). MCs are potent liver toxins and tumor promoters (Fujiki and Suganuma, 1994; Zhang et al., 2012; Tian et al., 2013; Wang et al., 2014, 2022; Hernandez et al., 2021). These cyanotoxins are extremely stable and are not effectively removed by conventional methods. They accumulate in aquatic animals and threaten human health and safety through the food chain (Zurawell et al., 2005; Zhang et al., 2009; Lance et al., 2010; Sotton et al., 2014; Pham and Utsumi, 2018; Kim et al., 2019; Zamora-Barrios et al., 2019). Human health risks caused by MCs may continue to increase in the future without effective intervening measures (Chen et al., 2005; Tian et al., 2013; Zhao et al., 2016, 2020; Janssen, 2019; Xiang et al., 2019; Weir et al., 2021; He et al., 2022). Consequently, it is essential to find a safe and effective MC treatment strategy.

Biodegradation is an especially promising means of MC removal (Wu et al., 2015; Li et al., 2017, 2021). Previous studies have reported many indigenous microorganisms in cyanobacterial bloom water. These microorganisms, which carry functional genes for specifically degrading cyanotoxins (Tsuji et al., 2006), include *Sphingomonas* sp. ACM-3962 (Bourne et al., 1996), *Sphingopyxis* sp. USTB-05 (Dexter et al., 2018), *Sphingopyxis* sp. IM-1 (Lezcano et al., 2016), *Sphingopyxis* sp. X20 (Qin et al., 2019), *Sphingopyxis* sp. YF1 (Yang et al., 2020), *Novosphingobium* sp. THN1 (Jiang et al., 2011), and *Novosphingobium* sp. ERW19 (Zeng et al., 2021). The genes involved in the MC degradation pathway, *mlr*A, *mlr*B, *mlr*C, and *mlr*D, together form the *mlr* gene cluster, which encodes the corresponding enzymes (Moron-Lopez et al., 2017; Zeng et al., 2020; Zhang et al., 2020). Unfortunately, not all genes of the *mlr* cluster can be successfully transcribed and expressed (Jiang et al., 2011). Therefore, it is necessary to separately study the heterogeneous expression and detailed catalytic mechanisms of these genes for furthering MC biodegradation. Among these enzymes, MlrA is responsible for the linearizing process of converting MCs to linearized MCs (Dexter et al., 2021). However, linearized MCs are still extremely toxic and must be further degraded (Wei et al., 2021). MlrB can degrade linearized MCs into tetrapeptides, but some reports show transcriptional silencing of the *mlr*B gene. This phenomenon is extremely unfavorable for the further detoxification of linearized MCs (Dziga et al., 2016; Li et al., 2022). Fortunately, the MlrC enzyme has dual degradation functions. MlrC can not only utilize the tetrapeptides obtained from the degradation of MCs by MlrB as substrate but also directly degrade the linearized MCs into the almost non-toxic Adda (Dziga et al., 2012). Therefore, it is essential to study the MlrC enzyme from different angles including the structural characteristics, degradation activity, biodegradation mechanism, and enzymatic improvements and modifications.

Knowledge of the molecular biodegradation processes of linearized MCs by the MlrC enzyme is limited. In previous studies on the molecular mechanism of MlrC, the MlrC structure had to be predicted first by homology modeling (Wei et al., 2021). The predicted structure may cause uncertainty in the mechanism studies. We obtained the actual three-dimensional structure of the MlrC enzyme derived from *Sphingomonas* sp. ACM-3962 in a previous study (PDB: 7YLQ), which laid the theoretical foundation for the further study of the catalytic mechanism. The MlrC enzyme is a type of metallopeptidase, and there is a zinc ion and four coordinated residues to form the catalytic center. The zinc ion plays a critical role in the catalytic activity of linearized MCs. There is also an empty position in the catalytic center next to the four residues and one water molecule. The empty position may be used for substrate binding in the subsequent degradation process. In addition, the two large domains of MlrC form a large central cavity for better accommodation of linearized heptapeptide substrates. Based on the actual three-dimensional structure of MlrC and its structural characteristics, we further explored the biodegradation molecular mechanism of the MlrC enzyme.

This study aimed to investigate the catalytic mechanism of the MlrC enzyme. The binding mode of MlrC and linearized MCs was obtained by the molecular docking method, and a series of key substrate-binding residues were found. They were further verified by site-directed mutagenesis. At the same time, the relationship between the MlrC enzyme (E), zinc ion (M), and substrates (S) was also studied in detail, and the components of the MlrC active center were explained. Finally, based on experimental studies and a literature survey, a possible catalytic mechanism was proposed. This study provided a better understanding of the catalytic mechanism of MlrC.

## 2. Materials and methods

### 2.1. Materials

Restriction enzymes Nde I and Xho I (Takara Biotech. Co. Ltd., Japan) were used to construct plasmids of wild-type MlrC and its variants. Standard MC-LR with purity ≥ 95% was purchased from Taiwan Algal Science Inc. (Taiwan, China) and stored at −20°C. Phosphoric acid and acetonitrile were purchased from TEDIA (HPLC/Spectro, United States) and used for high-performance liquid chromatography (HPLC) analysis. The expression vector pET21b and *E. coli* strain DH5α and BL21 (DE3) were purchased from Vazyme (Nanjing, China). Invitrogen Platinum SuperFi II DNA Polymerase, T4 DNA ligase, and restriction enzymes Nde I and Xho I were obtained from Thermo Fisher Scientific (United States).

### 2.2. Plasmid construction of MlrC and variants

MlrC and variant plasmids were constructed in *E. coli* strain BL21 (DE3) for protein overexpression and activity testing. The *mlr*C gene was PCR-amplified from genomic DNA derived from *Sphingomonas* sp. ACM-3962 and constructed into the pET21b vector (Invitrogen) with a C-terminal 6 × His tag. All primers used in this study are shown in Supplementary Table S2. Plasmid extraction was performed using the Tiangen Plasmid Mini Kit, and all clones were verified by DNA sequencing.

Site-directed mutagenesis was achieved by bridge PCR, and primers are listed in Supplementary Table S2, which generated the restriction enzyme sites NdeI and Xho1. pET21b-*mlr*C was used as the template for all mutants. The PCR program started at 95°C for 30 s, followed by 35 cycles of 58°C for 30 s and 72°C for 1.5 min. The plasmids containing the genes with a site-directed mutation were transferred into the *E. coli* strain BL21 (DE3). All mutants of pET21b-*mlr*C were verified by DNA sequencing.

### 2.3. Expression and purification of MlrC and variants

Transformed *E. coli* BL21 (DE3) containing pET21b-*mlr*C and the mutant vectors were cultured in liquid LB medium with 100 μg mL^−1^ ampicillin and shaken at constant conditions of 37°C and 220 rpm. When the optical density at 600 nm was approximately 0.6, isopropyl-β-D-thiogalactoside (IPTG) was added at a final concentration of 0.2 mM to induce protein expression. The induction conditions were 16°C, 210 rpm for 12–16 h. The *E. coli* cells were harvested by centrifugation (8,000 ×*g*, 10 min, 4°C), and the cells were resuspended in lysis buffer (Tris–HCl, pH 7.0, 150 mM NaCl). Cells were lysed by high-pressure cell disruption and then subjected to high-speed centrifugation (14,000 ×*g*, 60 min, 4°C). The crude enzyme solution was purified by Ni-NTA (Qiagen), washed with buffer B (25 mM Tris–HCl, pH 8.0, 150 mM NaCl, 15 mM imidazole), and eluted with buffer C (25 mM Tris–HCl, pH 8.0, 250 mM imidazole). The MlrC enzyme was characterized by sodium dodecyl sulfate–polyacrylamide gel electrophoresis (SDS-PAGE) on a 12% polyacrylamide gel. The concentration of purified MlrC protein was determined at 280 nm using an ultra-micro spectrophotometer (Nanodrop OneC, Thermo Fisher Scientific, United States).

### 2.4. Molecular docking calculations

Molecular docking of the linearized MC-LR to the MlrC structure was carried out with flexible zinc metalloprotein docking using AutoDock Vina. The crystal structure of MlrC was used as the receptor structure. The crystallized water molecules present in the structure and the metal ions (except the zinc ion in the active site) were removed. The ligand molecules of MlrC were prepared using Coot software, and before docking, the nonpolar H atoms were merged into both the ligands and the target by AutoDock Tools. Basic docking was performed by AutoDock Vina 1.1.2. The grid box was centered at x: 9.683, y: −36.275, and z: −27.867 with grid sizes of 80, 80, and 80 Å, respectively. Nine conformations were generated, and the best docking model of the linearized MC-LR with the lowest binding energy (−9.2 kcal mol^−1^) was selected and used as an evaluation standard for the subsequent calculations. Furthermore, the flexible zinc metalloprotein docking was also performed by AutoDock Vina 1.2.3. One zinc pseudo atom was added to the receptor. The grid box was centered at x: 7, y: −33, and z: −30 with grid sizes of 26.25, 31.5, and 24 Å, respectively. A total of 126 conformations were evaluated based on the proper distances of the hydrolysis site to the zinc ion, and the best conformation corresponded to the interactions between MlrC and the linearized MC-LR with a binding energy of −15.78 kcal mol^−1^ (from the AutoDock4 force field of AutoDock Vina 1.2.3). The docking method and processing of the linearized MC-RR and MC-YR were consistent with the linearized MC-LR. All figures representing the structures were generated by PyMOL (PyMOL Molecular Graphics System, Schrödinger, Inc.).

### 2.5. Enzyme activity assay

HPLC was used to determine the concentration of linearized MCs and their degradation products in samples. Linearized MCs were prepared by using MlrA to degrade standard MCs. The HPLC instrument was a DIONEX UltiMate 3000 (Thermo Fisher Scientific, United States) with a diode array detector equipped with an Acclaim™ 120 C18 column (4.6 mm × 250 mm, 5 μm particles, Thermo Fisher Scientific, Sunnyvale, United States). Linearized MCs and their degradation products were detected at a 238 nm wavelength and 1.0 mL min^−1^ flow rate. The injected volume was 50 μL, and the column temperature was 30°C. The mobile phase consisted of a gradient of a phosphoric acid solution (pH 3.84) containing 0.05% (V/V) of phosphoric acid (solvent A) and acetonitrile (solvent B). The gradient program was as follows: First, the column was balanced with 10% B for 3 min; then from 0 to 5 min, B was increased from 10 to 40%; from 5 to 12 min, B was increased from 40 to 70%; and from 12 to 12.5 min, B was decreased from 70 to 10%, and 10% B was used for 0.5 min. The linearized MC concentration was calculated using a linearized MC calibration curve method. The HPLC system had a detection limit of 0.1 μg L^−1^.

### 2.6. Analysis of circular dichroism spectra

Far-UV (190–260 nm) circular dichroism (CD) experiments for MlrC and variants of the zinc ion coordinated residues were carried out using a Chirascan CD Spectrometer (Applied Photophysics Ltd., Leatherhead, United Kingdom). Experiments were performed using solutions of protein at a concentration of 0.5 mg mL^−1^ in a 1 mm cell (Hellma UK Ltd., Southend, United Kingdom). Each CD spectrum represented the accumulation of three scans at 1 nm intervals with a 1.0 nm bandwidth and a time constant of 1 s. Protein secondary structure content was determined using CDNN version 2.1 (Institut für Biotechnologie, Martin-Luther Universität Halle-Wittenberg, Halle, Germany).

### 2.7. Fluorescence measurements

All fluorescence measurements were performed on a fluorescence spectrophotometer (Agilent Cary Eclipse, Malaysia) equipped with a xenon lamp source and a 1.0 cm quartz cell. Fluorescence emission spectra were recorded in the wavelength range of 285–420 nm upon an excitation wavelength of 280 nm. The excitation and emission bandwidth were 5 nm. The fluorescence quenching experiments of MlrC (1 μM) were performed at different concentrations of linearized MC-LR using a 1 cm path length fluorescence cuvette.

## 3. Results and discussion

### 3.1. The substrate-binding mode of MlrC

**Figure 1:**
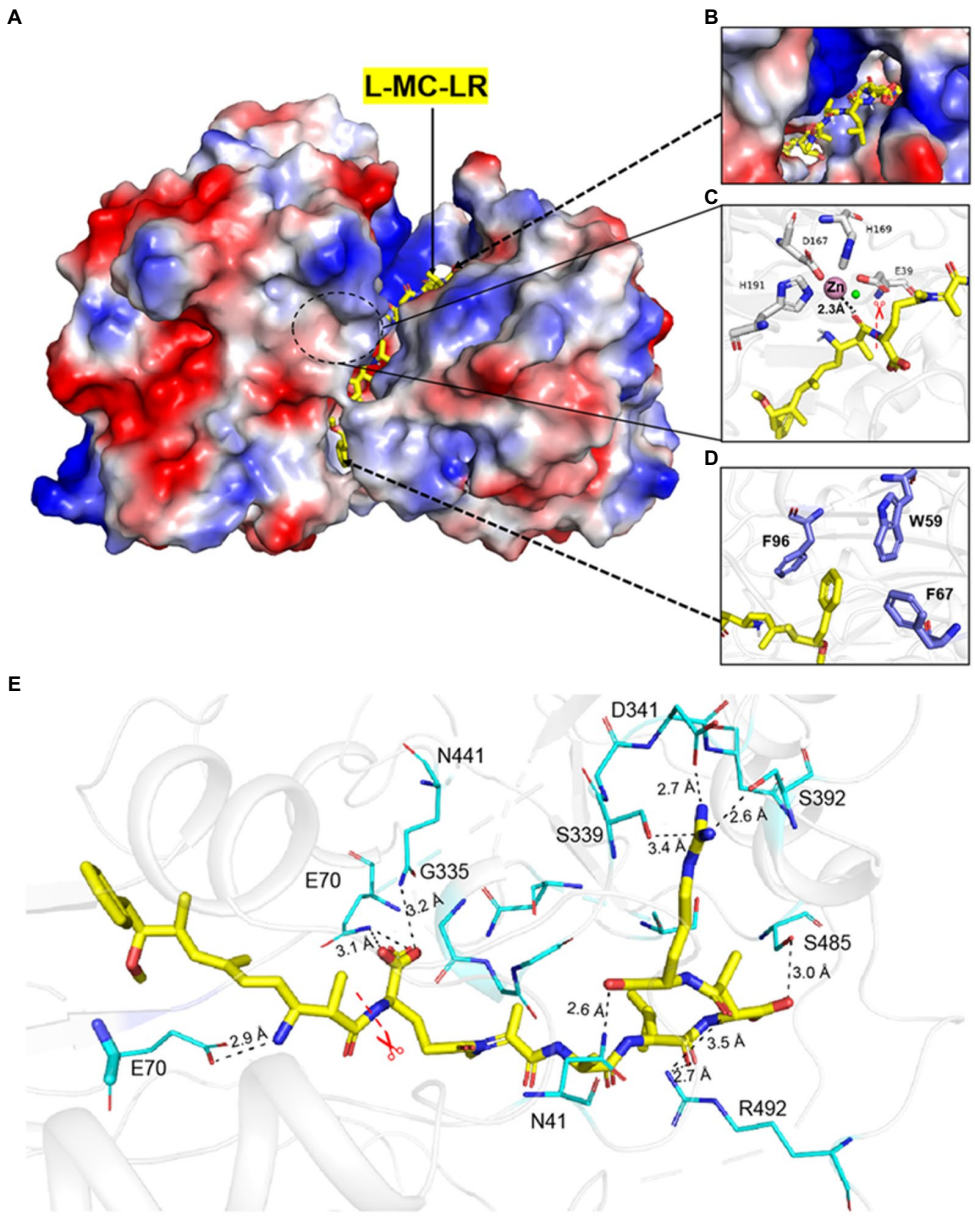
Interaction between MlrC and substrates. **(A–D)** The catalytic triad and the docking model of the reaction intermediate of linearized MC-LR in MlrC. **(E)** Residues involved in the binding site of MlrC. The MlrC structure is presented as a cartoon diagram in gray. Residues involved in binding linearized MC-LR are shown as a line model, and the amide bond that was cleaved by the enzyme is indicated with a star mark. The linearized MC-LR docking model is shown as a yellow stick figure. The hydrogen bonds formed between the residues and the substrate are shown as black lines.

To elucidate the substrate-binding mode of MlrC, we first attempted crystallography to obtain its complex structure with linearized MC-LR. However, neither co-crystallization nor soaking was successful on the complex, probably because its low solubility prevented us from using high concentrations of linearized MC-LR. Alternatively, we analyzed the substrate-binding mode by the molecular docking method using the crystal structure of MlrC (PDB: 7YLQ). The two large domains together formed a long and shallow cleft with dimensions of about 29 and 10 Å, which were used to accommodate the linearized substrates. The surface of the substrate-binding cleft was hydrophobic (Figures 1A,B). The amide bond of the linearized MC-LR cleavage site was close to the zinc ion, which was responsible for catalyzing the degradation of the substrate (Figure 1C).

The binding mode between the substrate and MlrC was mainly formed by the polar group of the linearized substrates. First, it was observed that E70 formed a hydrogen bond with an amine group of the linearized MC-LR (Figure 1E). Q468 formed a hydrogen bond with one of the carboxyl groups, and S485 and N41 formed a hydrogen bond with the other two carboxyl groups. R492 formed a hydrogen bond with a carbonyl, and D341 and S392 interacted with the terminal guanidine group of the linearized MC-LR. The hydrophobic regions formed by W59, F67, and F96 were used to accommodate the phenyl ring of the substrates and formed π–π interactions (Figure 1D). For the substrate-binding mode of MlrC and the linearized MC-RR and MC-YR, the key binding residues were similar to the linearized MC-LR. The only inconsistency was caused by the different characteristic groups between the three substrates (Supplementary Figures 2, 7). The residue R261 of MlrC was the binding site of the characteristic structure of linearized MC-RR. For the characteristic structure of linearized MC-YR, the binding site of MlrC was residue Y189 (Supplementary Figures 9, 10). The MlrC sequences of other microorganism species had high conservation according to sequence alignment, and this high conservation indicated excellent representativeness of the MlrC crystal structure from *Sphingomonas* sp. ACM-3962 (Supplementary Figure 3).

### 3.2. Enzyme activity of wild-type MlrC and variants

**Figure 2:**
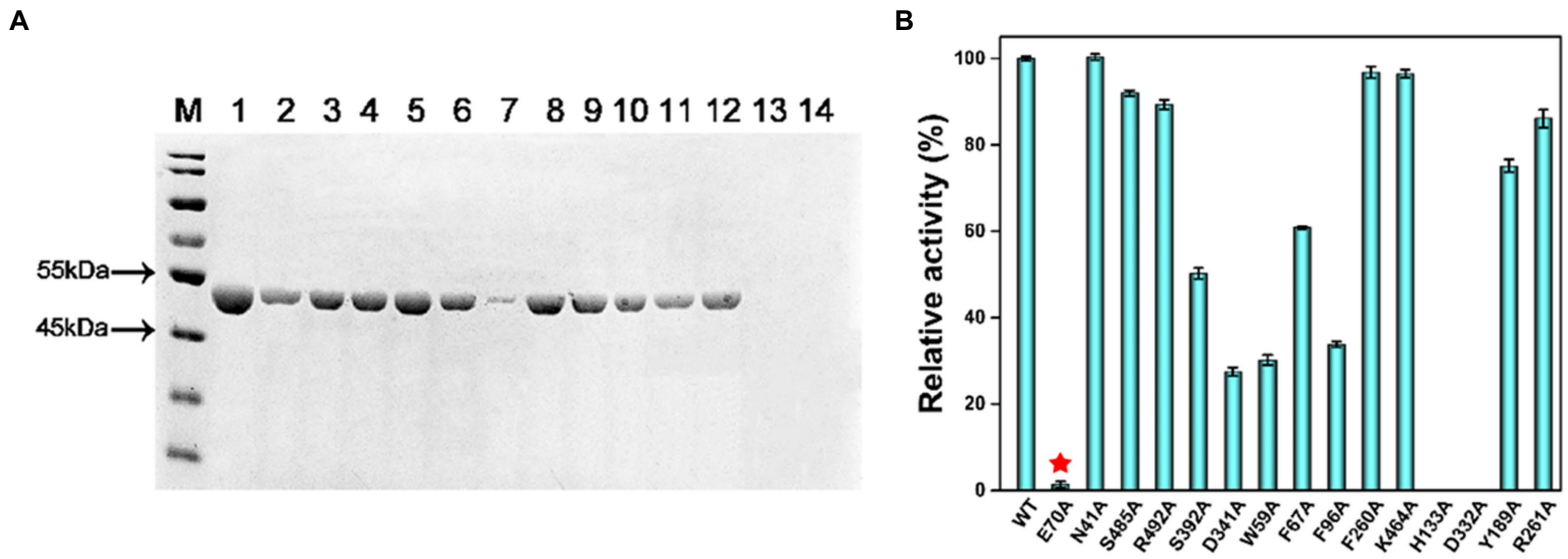
**(A)** Sodium dodecane sulfate–polyacrylamide gel electrophoresis (SDS-PAGE) was used to analyze samples of the variants for their substrate-binding residues. M, protein marker; Lane 1, wild-type MlrC; Lane 2, E70A; Lane 3, N41A; Lane 4, S485A; Lane 5, R492A; Lane 6, S392A; Lane 7, D341A; Lane 8, W59A; Lane 9, F67A; Lane 10, F96A; Lane 11, F260A; Lane 12, K464A; Lane 13, H133A; and Lane 14, D332A. **(B)** Hydrolytic activities of MlrC and its variants using linearized MC-LR as a substrate. Activities of MlrC and its variants were measured using a linearized MC-LR concentration of 0.25 mg L^−1^ and an enzyme concentration of 0.12 mg L^−1^. The amount of Adda produced was monitored by HPLC analysis. The activities of the variants were compared with wild-type MlrC.

Based on the analysis of the binding mode results by molecular docking, mutants were constructed, and their hydrolysis activities were measured. The activity of MlrC variants was reduced by different degrees (Figures 2A,B). It is worth noting that the *K*m and *K*cat values of E70 were both reduced. We suspected that E70 was not only responsible for binding with the linearized substrates but also involved in the catalytic process (Figure 2B; Table 1). F260, K464, H133, and D332 residues were mentioned by Wang et al. (2020), but no data were reported. We also tested the activity of the mutants, and the results showed that F260A and K464A did not significantly affect the activity of MlrC. However, H133A and D332A directly led to the unavailability of the MlrC enzyme, so we suspected that the two residues were critical for proper protein folding and stabilizing the structure of MlrC (Figure 2B; Supplementary Figure 11). All mutants were identified by circular dichroism experiments, except for the non-expressing mutants H133A and D332A. The results showed that the secondary structure of these mutants did not change, and the change in activity was caused by the destruction of the substrate-binding site (Supplementary Figure 6).

**Table 1:** Kinetic parameters of wild-type MlrC and variants.

Protein	*K*_m_ (μM)	*K*_cat_ (s − 1)	*K*_cat_/ *K*_m_ (s^−1^·μM^−1^)
MlrC ^WT^	0.24 ± 0.12	2409.98 ± 345.10	10041.48 ± 345.10
E70A	0.88 ± 0.55	1162.97 ± 397.00	1321.56 ± 397.00
N41A	0.65 ± 0.35	2276.20 ± 696.78	3501.85 ± 696.78
S392A	0.59 ± 0.10	2258.16 ± 203.15	3827.39 ± 203.15
S485A	0.47 ± 0.08	2072.21 ± 222.69	4406.83 ± 222.69
R492A	0.84 ± 0.24	2614.28 ± 420.62	3112.24 ± 420.62
D341A	0.81 ± 0.32	2730.61 ± 664.75	3371.12 ± 664.75
W59A	0.70 ± 0.22	2505.85 ± 460.63	3579.79 ± 460.63
F67A	0.70 ± 0.29	2549.51 ± 627.57	3642.16 ± 627.57
F96A	0.56 ± 0.10	2167.43 ± 216.98	3870.41 ± 216.98
F260A	0.25 ± 0.13	2464.32 ± 393.97	9857.28 ± 393.97
K464A	0.25 ± 0.14	2503.41 ± 485.77	10013.64 ± 485.77
D332A	-	-	-
H133A	-	-	-

### 3.3. Analysis of the relationship between the MlrC enzyme, zinc ion and substrate

Based on the analysis of the actual three-dimensional structure and substrate-binding mode of MlrC, we knew that the active center comprised the catalytic site and substrate-binding site. However, the relationship between the MlrC enzyme (E), zinc ion (M), and substrate (S) were not clear. Hence, their relationship was analyzed in detail in this study.

**Figure 3:**
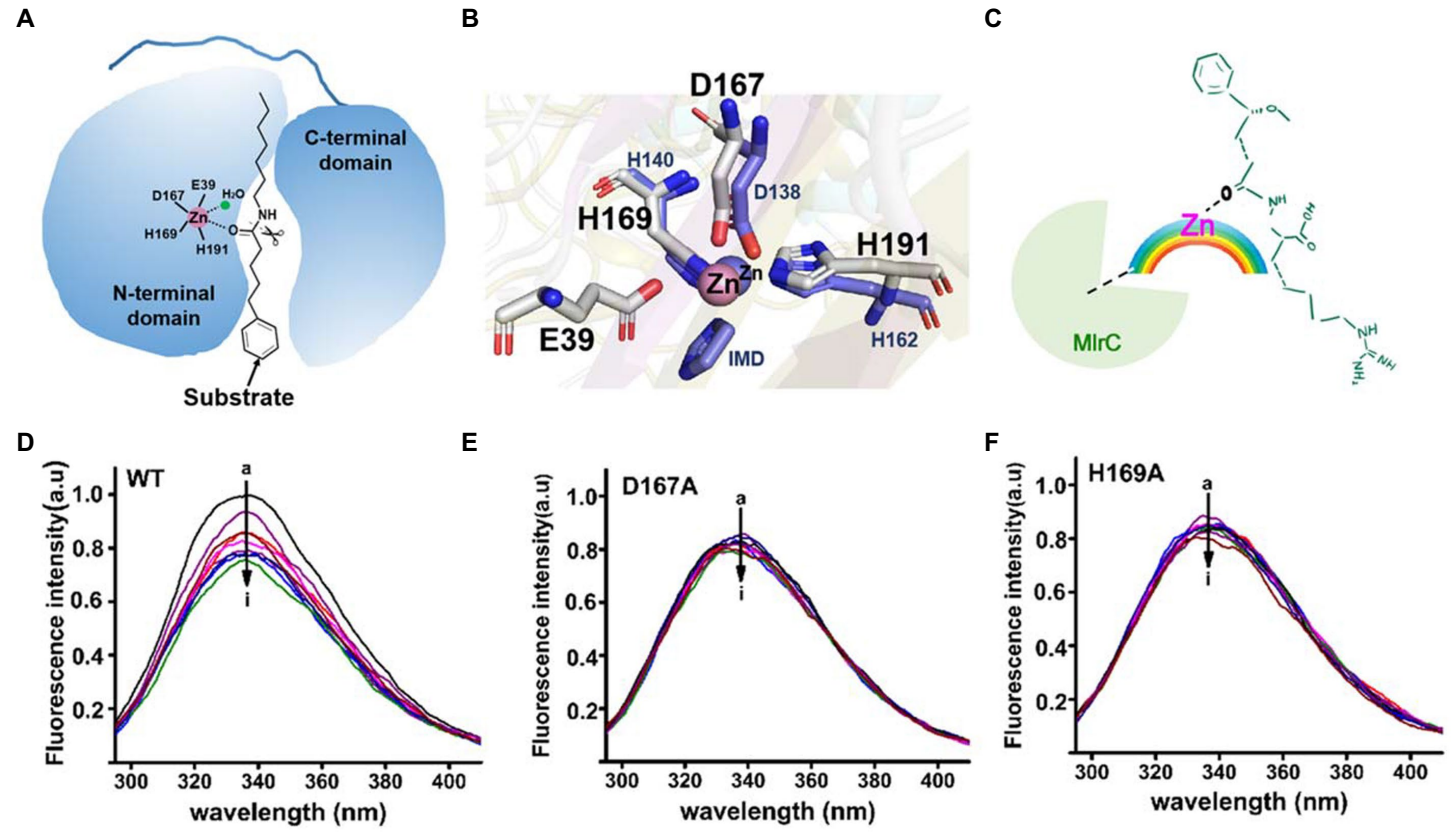
Analysis of the relationship between the MlrC enzyme, zinc ion, and linearized MC-LR. **(A)** Schematic model of the MlrC monomer structure and substrate. The two domains, the zinc ion with coordinated residues and the substrate model, are indicated. **(B)** Alignment of the zinc ion and coordinated residues between MlrC and 3IUU. **(C)** Schematic of the E-M-S model of the relationship between the MlrC enzyme, zinc ion, and linearized MC-LR, with the zinc ion acting as a metal bridge connecting the MlrC enzyme and substrate. **(D–F)** Synchronous fluorescence spectra of MlrC and variants: **(D)** wild-type MlrC; **(E)** D167A; and **(F)** H169A. The concentration of MlrC: 1 μM, and the concentration of linearized MC-LR a–i: 0, 1, 2, 3, 4, 5, 6, 7, and 8 μM, respectively.

The activity of MlrC was closely related to the zinc ion, which is located within the internal flexible region of the N-terminal catalytic domain to form the catalytic center. The crucial role of the zinc ion had been verified in our previous study. For the catalytic center of MlrC, the zinc ion coordinated with four residues, E39, D167, H169, and H196, to form a catalytic quadruplet, with a water molecule next to the zinc ion (Figure 3A). The zinc ion coordination condition of the homologous structure (PDB: 3IUU) was similar to that of MlrC, but the difference was that the zinc ion in 3IUU was coordinated by three residues and an exogenous imidazole group to form saturated coordination (Figure 3B). In contrast, the zinc ion in MlrC had four residues and one water molecule around it and left an unoccupied location for substrate binding. In conclusion, the zinc ion of MlrC was unsaturated coordination and left a redundant location that could be used to bind the substrates. Hence, the zinc ion of the catalytic center might bring MlrC and the substrate close to each other to guide the reactive group to the correct position (Figures 3A,B).

Although the zinc ion was essential for the MlrC activity, we questioned whether the absence of the zinc ion would affect MlrC and substrate binding. That is to say, the compositional relationship between the MlrC enzyme (E), zinc ion (M), and substrate (S) needed to be further explored. In this study, we used fluorescence spectroscopy experiments to research the relationship. Proteins have endogenous fluorescent properties. When the MlrC enzyme interacted with its specific substrates, the microenvironment around the fluorescent group in the MlrC enzyme changed, resulting in a decrease in the fluorescence intensity of the fluorescent molecule. If the MlrC enzyme could not interact with its substrates, the MlrC enzyme would not produce a significant fluorescence change. Therefore, mutants of residues D167 and H169, which made up the zinc ion catalytic center, were constructed. The wild-type MlrC was used as the control group, which could bind with substrates normally. The results showed that the two mutants D167A and H169A did not display an obvious change in fluorescence intensity, and only the wild-type MlrC did. We suspected that MlrC could bind the substrates normally, as it showed a change in fluorescence intensity. However, the D167A and H169A mutants directly prevented the formation of the zinc ion catalytic center, so the substrate and MlrC were not able to bind correctly, resulting in no significant change in fluorescence intensity (Figures 3D–F). Combined with the above analysis, we speculated that the MlrC enzyme (E), substrate (S), and zinc ion (M) formed E-M-S intermediates during the catalytic process of MlrC. The zinc ion acted as a metal bridge, which brought MlrC and the substrates closer to each other and guided the reactive group of the substrates into the correct position (Figure 3C). Therefore, MlrC and the substrates could not bind once the zinc ion was absent.

### 3.4. Linearized MC degradation mechanism by MlrC

**Figure 4:**
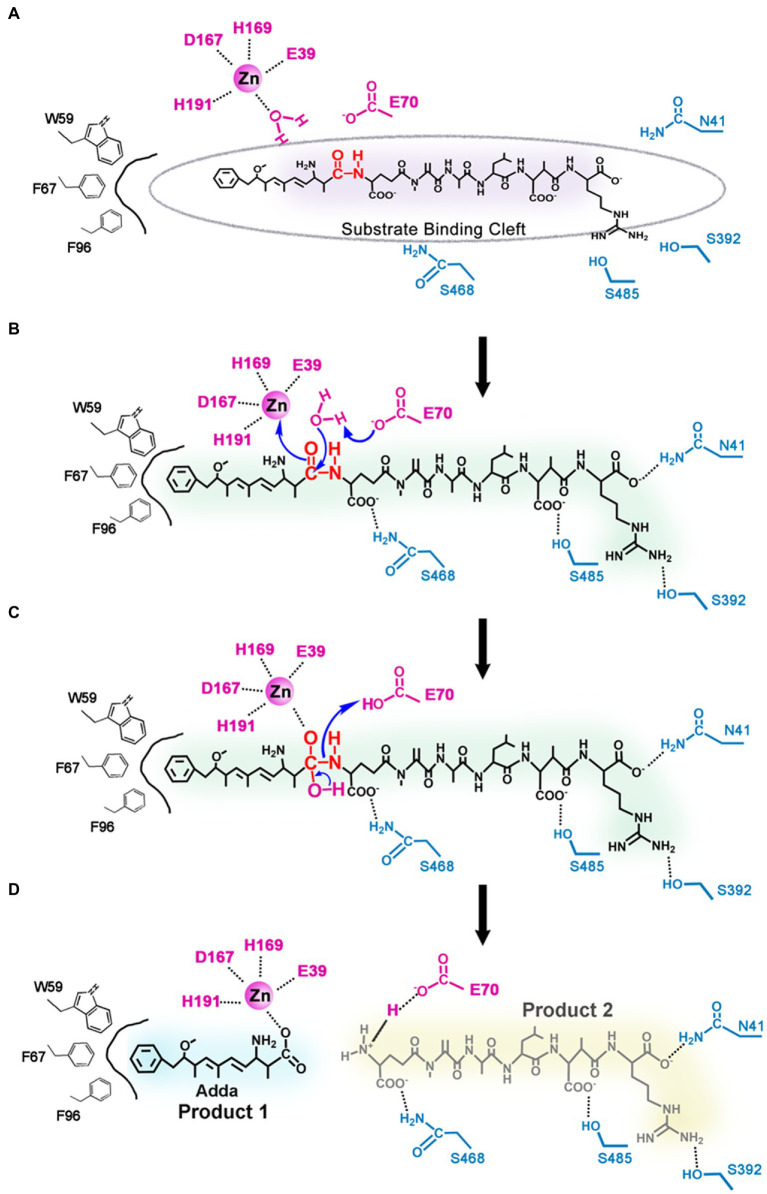
Proposed catalytic mechanism of linearized MC-LR biodegradation by MlrC. **(A)** The MlrC active center is composed of the catalytic site (the zinc ion, one water molecule, and the residues E39, D167, H169, and H191) and the substrate-binding site (the residues E70, W59, F67, F96, N41, S392, S485, and S468); **(B)** Residue E70 as a general base catalyst removing a proton of the metal-bound water to form zinc-OH-nucleophile for attacking the amide bond of the substrate. **(C)** E70 as a general acid catalyst donating a proton to the amine. **(D)** Two products of linearized MC-LR biodegradation by MlrC.

Based on the results of the molecular docking and biochemical experiments, a possible catalytic mechanism of MlrC was proposed (Figure 4). The MlrC active center was mainly composed of two parts, the catalytic site and the substrate-binding site. The catalytic site was composed of the zinc ion and the four coordinated residues E39, D167, H169, and H191, with one water molecule involved in catalysis. The coordination layer of the zinc ion was not saturated, leaving an extra position that combined with the substrate to form a saturated coordination layer. The enzyme (E), substrate (S), and metal ion (M) formed E-M-S intermediates during the catalytic process. The zinc ion acted as a metal ion bridge, making the distance between MlrC and the substrates closer, which is conducive to MlrC acting on the reactive group of the substrates. This meant that the MlrC enzyme and its substrate could not be combined once the metal ion was absent. This was verified by fluorescence spectroscopy experiments (Figures 3D–F). The substrate-binding cavity was made up of N-and C-terminal domains (Figure 3A), and the substrate-binding site mainly included N41, E70, D341, S392, Q468, S485, R492, W59, F67, and F96. These residues were verified by site-directed mutagenesis and activity test experiments. Among them, the E70 residue was very critical. On the one hand, this residue was responsible for forming hydrogen bonds with one amine group of the substrate. On the other hand, it was responsible for activating the H_2_O molecule, which was next to the zinc ion to form zinc-OH-nucleophile to attack the substrate cleavage site. Activity experiments also showed that the activity of residue E70A was significantly reduced (Figure 3C; Table 1).

During the entire catalytic process, residue E70 acted as a general base catalyst by removing a proton from the metal-bound water in the first step, allowing the metal-bound hydroxide to attack the carbonyl group of the substrate peptide, while the negative charge of E39 stabilized the transition state (Figure 4A). The other substrate-binding residues, N41, D341, S392, Q468, S485, R492, W59, F67, and F96, played a role in binding substrates and helped locate the substrate to be catalyzed. In the next step, E70 acted as a general acid catalyst by donating a proton to the leaving amine (Figure 4B), which eventually led to the collapse of the metal-bound tetrahedral intermediate (Figure 4C). The N-terminal product left and water returned to the metal ion, while the C-terminal product was still bound to E70 through a salt bridge. Through a series of reactions, the substrate peptide bond was finally hydrolyzed (Figure 4D). In conclusion, the nucleophilic attack on the substrate cleavage site was mediated by the water molecule, and the water molecule cooperated with the metal ion-zinc ion coordinated by multiple residues to complete the catalytic process of MlrC.

## 4. Conclusion

Through molecular docking and site-directed mutagenesis methods, the binding mode of MlrC with linearized MC-LR was studied. A series of key substrate-binding residues were identified. Among them, residue E70 was very specific and crucial for the substrate degradation process by the MlrC enzyme. E70 formed a hydrogen bond with one amine group of the substrates. More importantly, E70 was responsible for activating the water molecule near the zinc ion to attack the cleavage site of the substrates. The importance of residue E70 was verified by biochemical experiments. Other substrate-binding residues were also evaluated by site-directed mutagenesis. Based on the analysis of the active center formed by the catalytic site and substrate-binding site, the relationship between the MlrC enzyme (E), zinc ion (M), and substrate (S) was analyzed in detail. They formed an E-M-S intermediate during the catalytic process. The zinc ion acted as a metal ion bridge, making the distance between MlrC and the substrates closer, which is conducive to MlrC acting on the reactive group of the substrates. Eventually, the catalytic degradation mechanism of MlrC was proposed. The nucleophilic attack on the substrate cleavage site was mediated by the water molecule, and the zinc ion assisted in the catalytic process, which is coordinated with multiple residues.

In conclusion, MlrC effectively promoted the degradation of linearized MCs, which play a unique role in detoxifying MCs. This study lays a foundation for the biodegradation mechanism of linearized MCs. MlrC may be useful for the complete elimination of cyanotoxins. This is of great significance for the research and application of MCs decontamination. Finally, how to apply the MC-specific enzymes to cyanotoxins degradation in polluted water is worthy of further study.

## Data availability statement

The original contributions presented in the study are included in the article/Supplementary material, further inquiries can be directed to the corresponding authors.

## Author contributions

XG, LF, and WD conceived and designed all experiments. XG performed protein purification and crystallization. XG, ZL, and QJ analyzed the data. XG, QJ, CC, YF, YH, and LZ performed the biochemical assays. XG and LF wrote and revised the paper. All authors contributed to the article and approved the submitted version.

## Funding

This study was supported by the National Natural Science Foundation of China (grant numbers 22277037, 21877046, 21472061, and 21272089), the Fundamental Research Funds for the Central Universities (grant numbers CCNU18ZDPY02, CCNU18TS010, CCNU16A02041, and CCNU14A05006), and the Program of Introducing Talents of Discipline to Universities of China (111 Program, B17019).

## Conflict of interest

The authors declare that the research was conducted in the absence of any commercial or financial relationships that could be construed as a potential conflict of interest.

## Publisher’s note

All claims expressed in this article are solely those of the authors and do not necessarily represent those of their affiliated organizations, or those of the publisher, the editors and the reviewers. Any product that may be evaluated in this article, or claim that may be made by its manufacturer, is not guaranteed or endorsed by the publisher.
