# Supplementary material for: Structural insight into the substrate-binding mode and catalytic mechanism for MlrC enzyme of Sphingomonas sp. ACM-3962 in linearized microcystin biodegradation
Source: Front Microbiol. 2023 Feb 17;14:1057264. doi: 10.3389/fmicb.2023.1057264 (PMC9982164; doi:10.3389/fmicb.2023.1057264)
Supplement: Supplementary file 1 [file Data_Sheet_1.PDF]

Supplementary Material

S1. Supporting Methods and Materials

S1.1 Compare MlrC structure against previously known protein structures

Dali Server (Protein Structure Database Searching by DaliLite v.3) is a web service for comparing protein structures in 3D. Dali can compare the new structure to previously known protein structures. If significant similarities are found, it may indicate distant homology, i.e. the structures share a common origin. This may be important in identifying molecular mechanisms that may remain very similar from distant predecessors to the present.

http://ekhidna2.biocenter.helsinki.fi/dali/oldstyle.html

S1.2 Molecular Dynamics (MD) Simulations of MlrC Complex with Linearized MC-LR

To confirm the binding mode of L-MC-LR, MD simulations were performed by GROMACS (Abraham et al., 2015; Bauer et al., 2022a; Bauer et al., 2022b;), based on the MlrC complex with L-MC-LR. The protein parameters were used by CHARMM36 force field (Best et al., 2012), whereas the parameters of L-MC-LR were solved by CGenFF server (Vanommeslaeghe et al., 2010). The Na (I) ions were used to neutralize the system, and then an octahedral box of TIP3P water molecules, which extended 20 Å from any given atom of MlrC, were selected for solvation. The MD was simulated with constant pressure and temperature (1 bar at 300 K) at periodic boundary conditions. The ligand was restrained by generating a position restrain topology. Equilibration was monitored by combining the stability of the temperature, energies and densities of the system, as well as the rmsd values of the backbone atoms.

Reference

Abraham, M. J., Murtola, T., Schulz, R., S Páll, Smith, J. C., Hess, B., Lindahl, E., 2015. Gromacs: high performance molecular simulations through multi-level parallelism from laptops to supercomputers. SoftwareX.

Bauer, P., Hess, B., Lindahl, E., 2022. GROMACS 2022.2 Manual.

Bauer, P., Hess, B., Lindahl, E., 2022. GROMACS 2022.2 Source code.

Best, R. B., Xiao, Z., Shim, J., Lopes, P., Mackerell, A. D., 2012. Optimization of the Additive CHARMM All-Atom Protein Force Field Targeting Improved Sampling of the Backbone ϕ, ψ and Side-Chain $\chi 1$ and $\chi 2$ Dihedral Angles. Journal of Chemical Theory and Computation 8, 3257-3273.

Vanommeslaeghe, K.,Hatcher, E., Acharya, C., Kundu, S., Zhong, S., Shim, J., Darian, E., Guvench, O., Lopes, P., Vorobyov, I., Mackerell, A D., 2010. CHARMM General Force Field: A Force Field for Drug-Like Molecules Compatible with the CHARMM All-Atom Additive Biological Force Fields. J Comput Chem **31**, 671-690.

Table S1. Dali search results of the MlrC structure

chain	Z-score	RMSD (Å)	Residue number	identity (%)	Description
3IUU-A	40.2	2.5	494	23	Putative metallopeptidase
4ry0-A	10.2	3.5	287	8	Probable ribose abc transporter, substrate-bindin
4wt7-A	10.0	4.1	294	13	ABC transporter substrate binding protein (ribose)
2fn8-A	9.7	3.8	292	11	Ribose abc transporter, periplasmic ribose-bindin
4ry9-A	9.5	4.4	297	16	Periplasmic binding protein/laci transcriptional
2rjn-A	9.4	2.8	135	11	Pesponse regulator receiver:metal-dependent
4l4u-A	9.4	17.5	388	11	Transcriptional regulator (ntrc family)
4yv7-A	9.3	4.1	288	11	Periplasmic binding protein/laci transcriptional
5m7o-A	9.3	17.4	448	9	Nitrogen assimilation regulatory protein
4wzz-A	9.2	3.5	321	7	Putative sugar abc transporter, substrate-binding
4rk0-D	9.1	3.4	269	8	Laci family sugar-binding transcriptional regulate
4rwe-A	9.0	4.4	287	12	Sugar-binding transport protein

Table S2 Primers used in the construction of recombinant plasmids and mutagenesis.

Primer	Sequence (5' to 3')	Amino acid
mlrC-F	ATACATATGCTGGACCGTCGTACCC	1-507
mlrC-R	GCCCTCGAGAACCGGTTCGAACGGCC	1-507
N41A-F	GTACCGAAACCGCATCTTTCTCTCC	1-507
S485A-F	CTACGTTGCGGCATCTCAGCAGTC	1-507
R492A-F	AGTCTTCTATCGCACACTACCACAA	1-507
S392A-F	GTGAAGCGGCAGGTCTGCC	1-507
D341A-F	TGCGTCTGGTGCAAACATGGCG	1-507
W59A-F	CCATGCTGGCACGTCCGGG	1-507
F67A-F	ACACCCGGACGCAGCGACCGA	1-507
F96A-F	ACCTGCGCGGCAGCGATGCCGGG	1-507
E70A-F	TTCGCGACCGCAGCGACCGGT	1-507
H133A-F	GGTCTGGCCGGTGCGAT	1-507
D332A-F	GACTCTTCTGCCAACCCGGGT	1-507
F260A-F	ATCCAGGGTGCTCGTGCGGGT	1-507
K464A-F	ACGTTGCGGTTGCTTCTTCTGAACA	1-507
R261A-F	CAGGGTTTCGCAGCGGGTGAC	1-507
Y189A-F	GTTCAAATACGCACCGCACATC	1-507

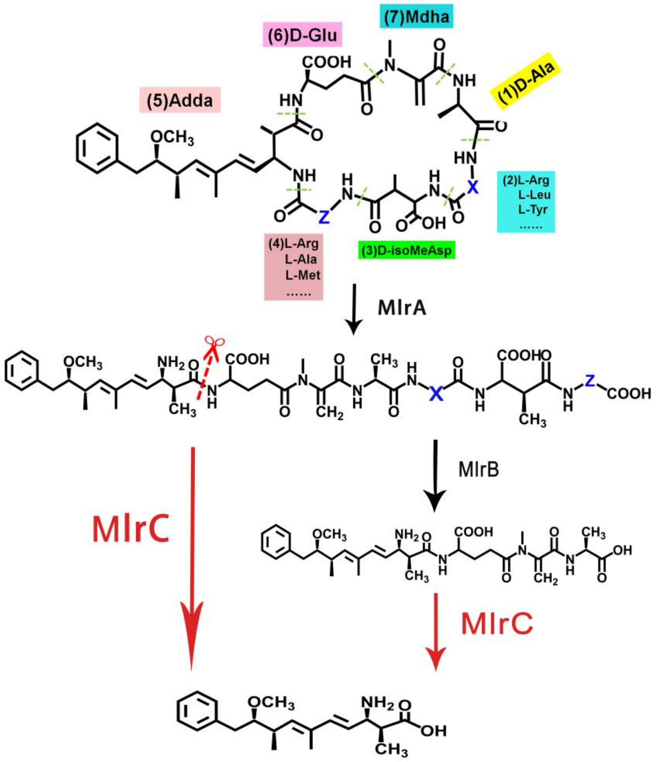

Supplementary Figure 1. Structures of MlrC hydrolysis products.

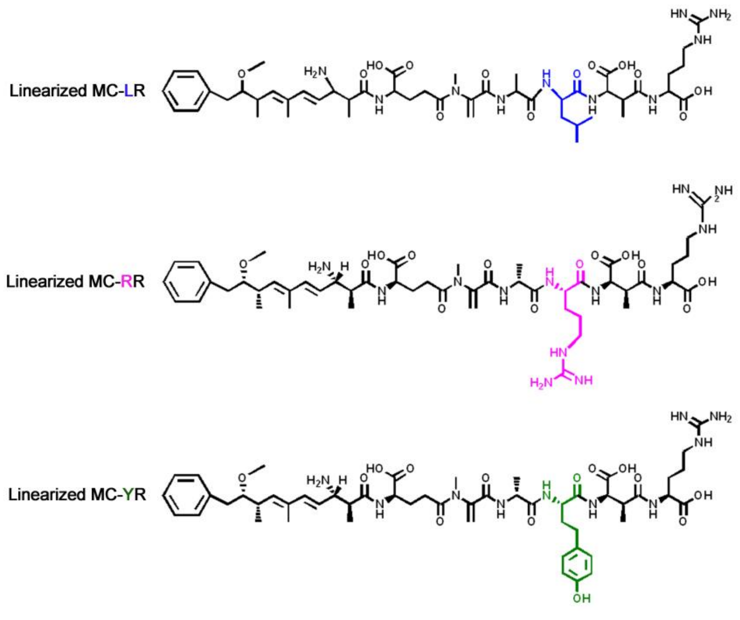

Supplementary Figure 2. The different characteristic groups between three substrates linearized MC-LR/MC-RR/MC-YR of MlrC.

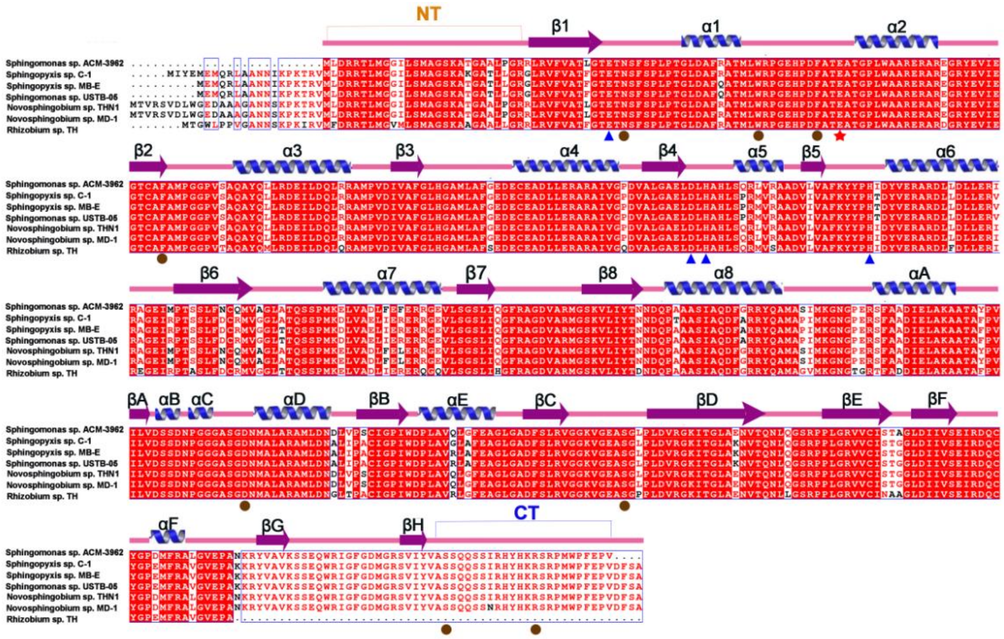

Supplementary Figure 3. Multiple sequence alignment (generated by Multalin) displays MlrC from *Sphingomonas* sp. ACM-3962 (Gene Bank: AF411070.1) and six homologues: *Sphingopyxis* sp. C-1 (Gene Bank: BAI47772.1), *Sphingopyxis* sp. MB-E (Gene Bank: AOW44969.1), *Sphingomonas* sp. USTB-05 (Gene Bank: AGH62481.1), *Novosphingobium* sp. THN1 (Gene Bank: AEC46645.1), *Novosphingobium* sp. MD-1 (NCBI Reference Sequence: WP_125955356.1), *Rhizobium* sp. TH (GenBank: AQV08233.1). (The blue stars represent the coordinated residues with zinc ion of catalytic center, the brown circles and red star represent the key substrate binding sites).

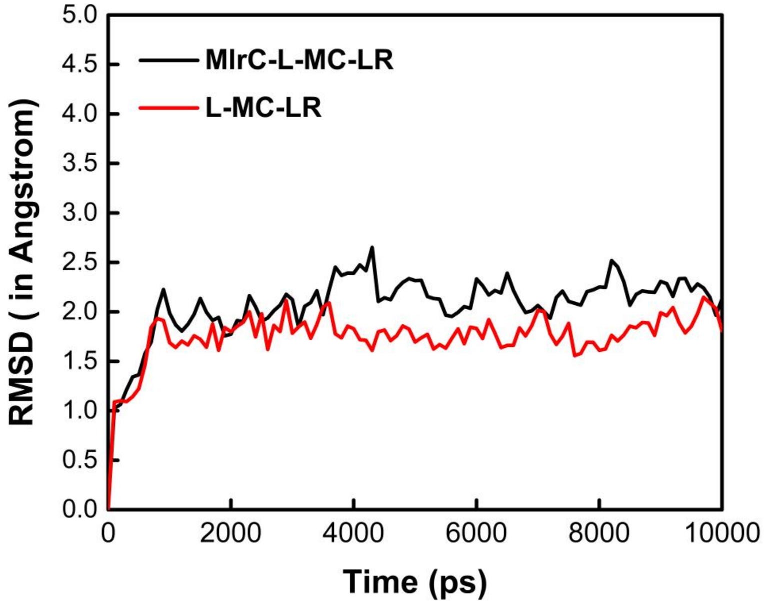

Supplementary Figure 4. Plots of the molecular dynamic (MD) simulation time vs root-mean-square deviation (rmsd, in Å) of the all atoms of the MlrC in complex with substrate linearized MC-LR (abbreviated as L-MC-LR). The black line represents the rmsd of the entire enzyme and L-MC-LR, the red line represents the corresponding rmsd of the L-MC-LR.

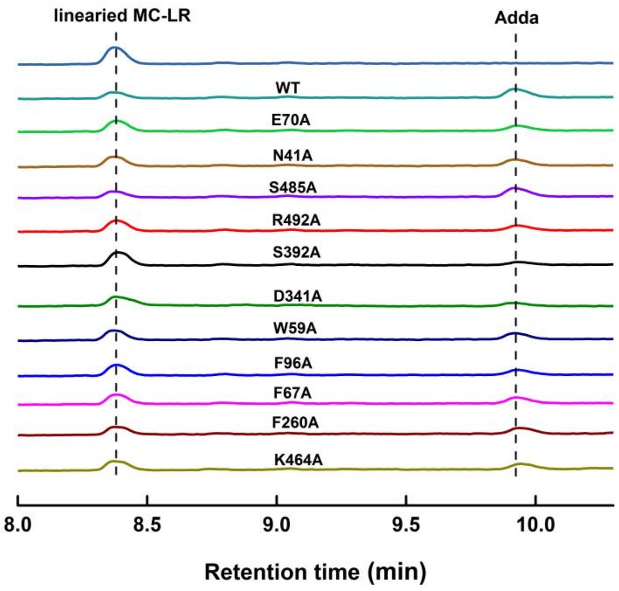

Supplementary Figure 5. HPLC spectrogram of MlrC wild type and its variants for the key substrate binding sites.

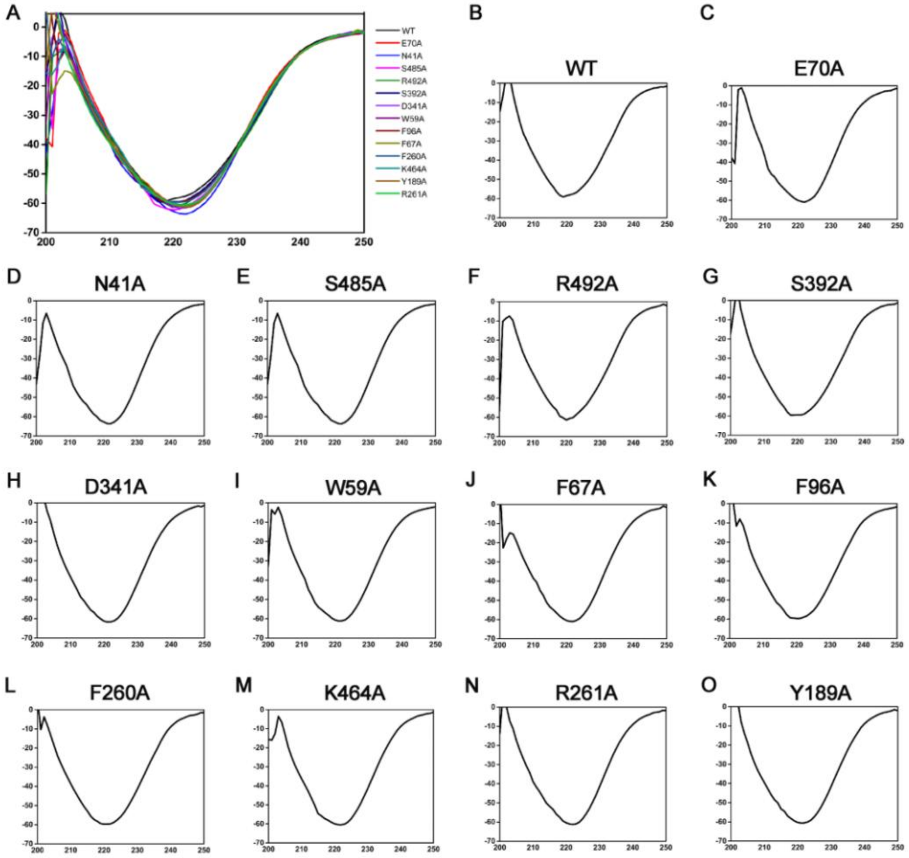

Supplementary Figure 6. Far-UV Circular dichroism. (A) Superposed CD spectra of MlrC wild type and its variants for the key substrate binding sites. (B)-(O) Each spectrum of the variants is also

shown in below. The spectra indicate that there is no significant difference among the variants and MlrC wild type.

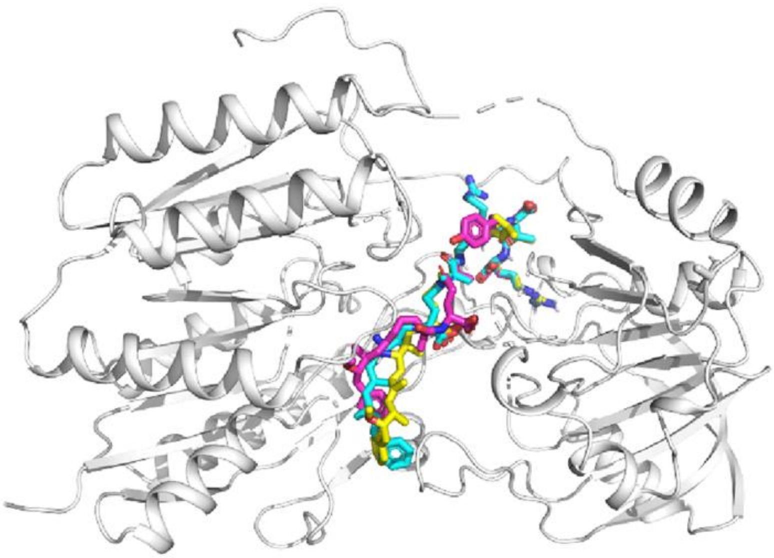

Supplementary Figure 7. Alignment of binding mode for MlrC and three substrate linearized MC-LR/MC-RR/MC-YR.

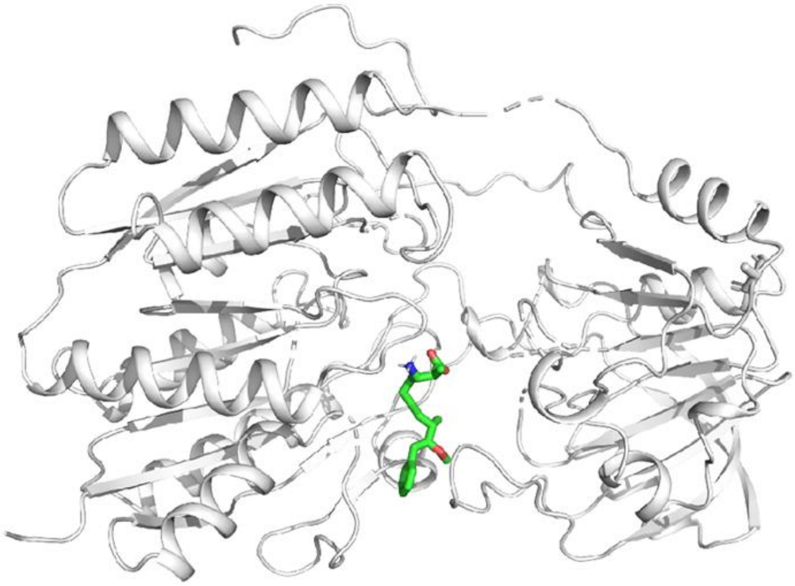

Supplementary Figure 8. Binding mode of MlrC and product Adda.

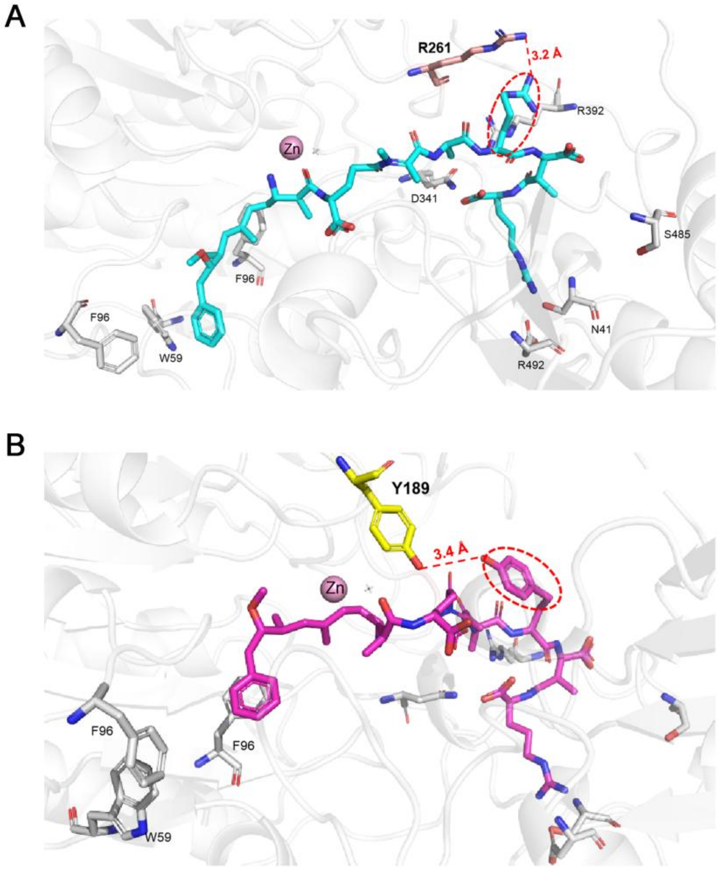

Supplementary Figure 9. MlrC features binding sites with linearized MC-RR and linearized MC-YR compared to linearized MC-LR.

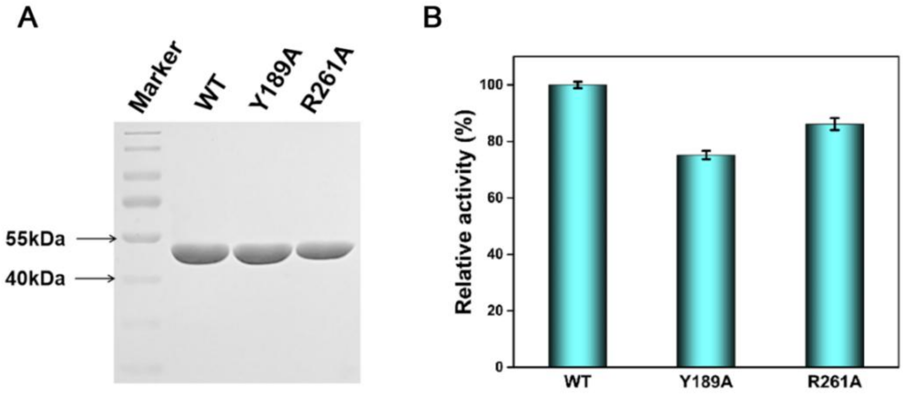

Supplementary Figure 10. (A) Sodium dodecane sulfate polyacrylamide gel electrophoresis (SDS-PAGE) of variants for substrate-binding sites of linearized MC-RR/MC-YR. M, protein marker; Lane Y189A, the variant of binding site for characteristic groups in linearized MC-RR; Lane R261A, the variant of binding site for characteristic groups in linearized MC-YR. (B) Hydrolytic activities of MlrC and its variants using linearized MC-RR and linearized MC-YR as substrates. MlrC activities and its variants were measured using linearized MC-LR/MC-YR concentration of 0.25 mg L^{-1} and enzyme concentration of 0.12 mg L^{-1}. The amount of produced Adda was monitored by HPLC analysis. The activitie of the variants were compared with WT of MlrC.

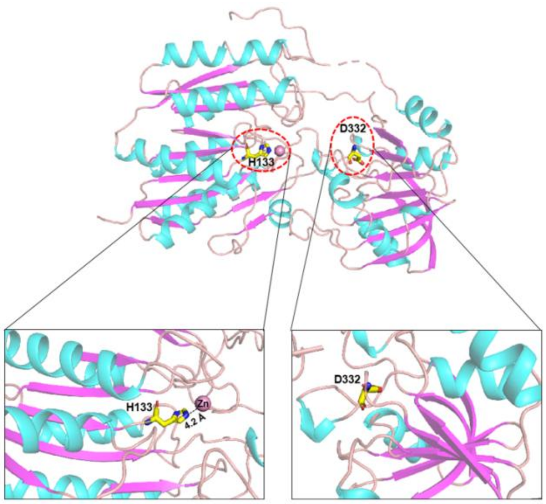

Supplementary Figure 11. The positions of residues D332 and H133 in the three-dimensional structure of MlrC.

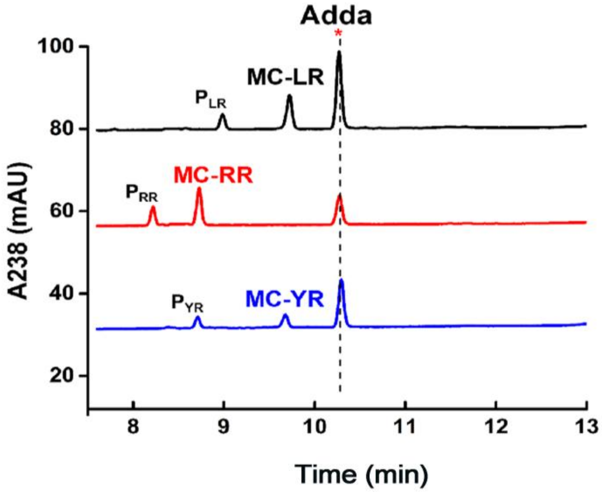

Supplementary Figure 12. HPLC chromatogram of Adda product with retention times 10.4 min at 238 nm after degradation by MlrC. P_{LR}, P_{RR}, and P_{YR} represent linearized microcystins, which produced from acyclic MCs (MC-LR/MC-RR/MC-YR). The spectra indicate that MlrC can degrade the three most widely distributed and toxic linearized MC-LR.

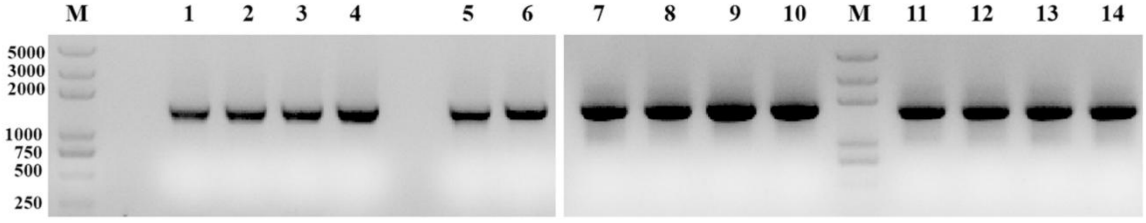

Supplementary Figure 13. PCR products of MlrC-WT and all variants for substrate-binding sites. The length of *mlr*C gene is 1521bp. M, protein marker; Lane 1, wild type of MlrC; Lane 2, E70A; Lane 3, N41A; Lane 4, S485A; Lane 5, R492A; Lane 6, S392A; Lane 7, D341A; Lane 8, W59A;

Lane 9, F67A; Lane 10, F96A; Lane 11, F260A; Lane 12, K464A; Lane 13, H133A; Lane 14, D332A.
